# Neurocan, an extracellular chondroitin sulfate proteoglycan, stimulates neuroblastoma cells to promote malignant phenotypes

**DOI:** 10.18632/oncotarget.22435

**Published:** 2017-11-15

**Authors:** Zhendong Su, Satoshi Kishida, Shoma Tsubota, Kazuma Sakamoto, Dongliang Cao, Shinichi Kiyonari, Miki Ohira, Takehiko Kamijo, Atsushi Narita, Yinyan Xu, Yoshiyuki Takahashi, Kenji Kadomatsu

**Affiliations:** ^1^ Department of Biochemistry, Nagoya University Graduate School of Medicine, Nagoya, Aichi, Japan; ^2^ Research Institute for Clinical Oncology, Saitama Cancer Center, Saitama, Saitama, Japan; ^3^ Department of Pediatrics, Nagoya University Graduate School of Medicine, Nagoya, Aichi, Japan

**Keywords:** NCAN, CSPG, neuroblastoma, tumor sphere

## Abstract

Neurocan (NCAN), a secreted chondroitin sulfate proteoglycan, is one of the major inhibitory molecules for axon regeneration in nervous injury. However, its role in cancer is not clear. Here we observed that high NCAN expression was closely associated with the unfavorable outcome of neuroblastoma (NB). NCAN was also highly and ubiquitously expressed in the early lesions and terminal tumor of TH-*MYCN* mice, a NB model. Interestingly, exogenous NCAN (i.e., overexpression, recombinant protein and conditioned medium) transformed adherent NB cells into spheres whose malignancies *in vitro* (anchorage-independent growth and chemoresistance) and *in vivo* (xenograft tumor growth) were potentiated. Both chondroitin sulfate sugar chains and NCAN's core protein were essential for the sphere formation. The CSG3 domain was essential in the moiety of NCAN. Our comprehensive microarray analysis and RT-qPCR of mRNA expression suggested that NCAN treatment promoted cell division, and urged cells to undifferentiated state. The knockdown of NCAN in tumor sphere cells cultured from TH-*MYCN* mice resulted in growth suppression *in vitro* and *in vivo*. Our findings suggest that NCAN, which stimulates NB cells to promote malignant phenotypes, is an extracellular molecule providing a growth advantage to cancer cells.

## INTRODUCTION

Neuroblastoma (NB), which originates from the sympathoadrenal lineage of the neural crest in humans, is the most common extracranial solid tumor in infancy. [1]. The differentiated status of NB cells is closely related to malignancy: the more primitive and neural crest-like histologies cause worse prognoses [2]. The elucidation of molecules and signaling pathways involved in the suppression of neuronal differentiation will thus clarify the mechanisms underlying NB progression and could suggest specific interventions for NB.

Proteoglycans, one of the major components of the extracellular matrix, play important roles in the phenomena such as cell motility, development, differentiation and the maintenance of stemness [3–6]. The major extracellular proteoglycans are classified into chondroitin sulfate proteoglycans (CSPGs), dermatan sulfate proteoglycans (DSPGs), keratan sulfate proteoglycans (KSPGs), and heparan sulfate proteoglycans (HSPGs). Each glycan chain is attached to particular core proteins [7]. Among these proteoglycans, CSPGs and HSPGs have been well studied, and it was shown that CSPGs and HSPGs exert completely opposite effects, suppressive or promoting axon regeneration, respectively [8]. It was reported that HSPGs were involved in the regulation of NB cells. HSPGs promote the differentiation of neuroblasts, and they suppress NB growth [9, 10]. On the other hand, the roles of CSPGs in NB pathogenesis have not been studied yet.

The CSPG neurocan (NCAN) is a member of the lectican family, which includes aggrecan (ACAN), versican (VCAN) and brevican (BCAN). NCAN is expressed mainly in nervous tissues [11]. Consistent with the activity of CSPG mentioned above, it was reported that NCAN expression was up-regulated in a nerve injury region, and that nerve regeneration and sensory neuron extension were inhibited through protein tyrosine phosphatase receptor sigma (PTPRσ) [8, 12]. To the best of our knowledge, the roles of NCAN in cancer have not been investigated. Here we report the ability of NCAN to induce an undifferentiated phenotype, and its ability to promote the malignancy of NB cells.

## RESULTS

### A high expression of NCAN is closely associated with unfavorable outcomes of NB patients

**Figure 1:**
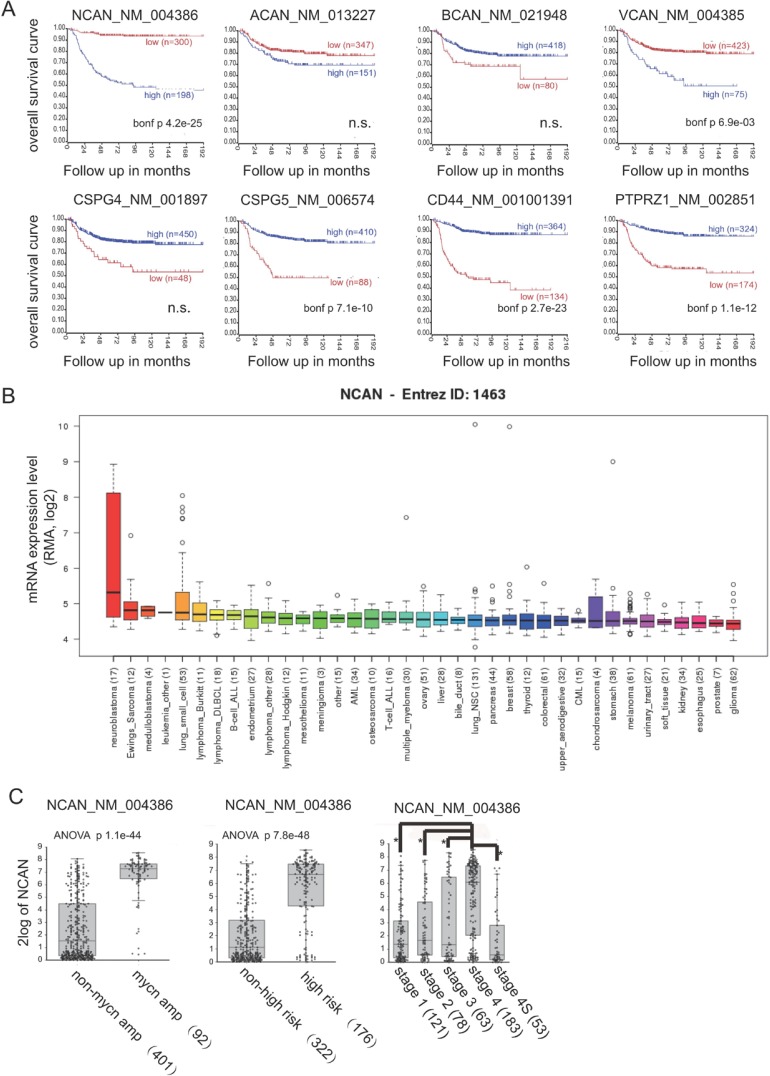

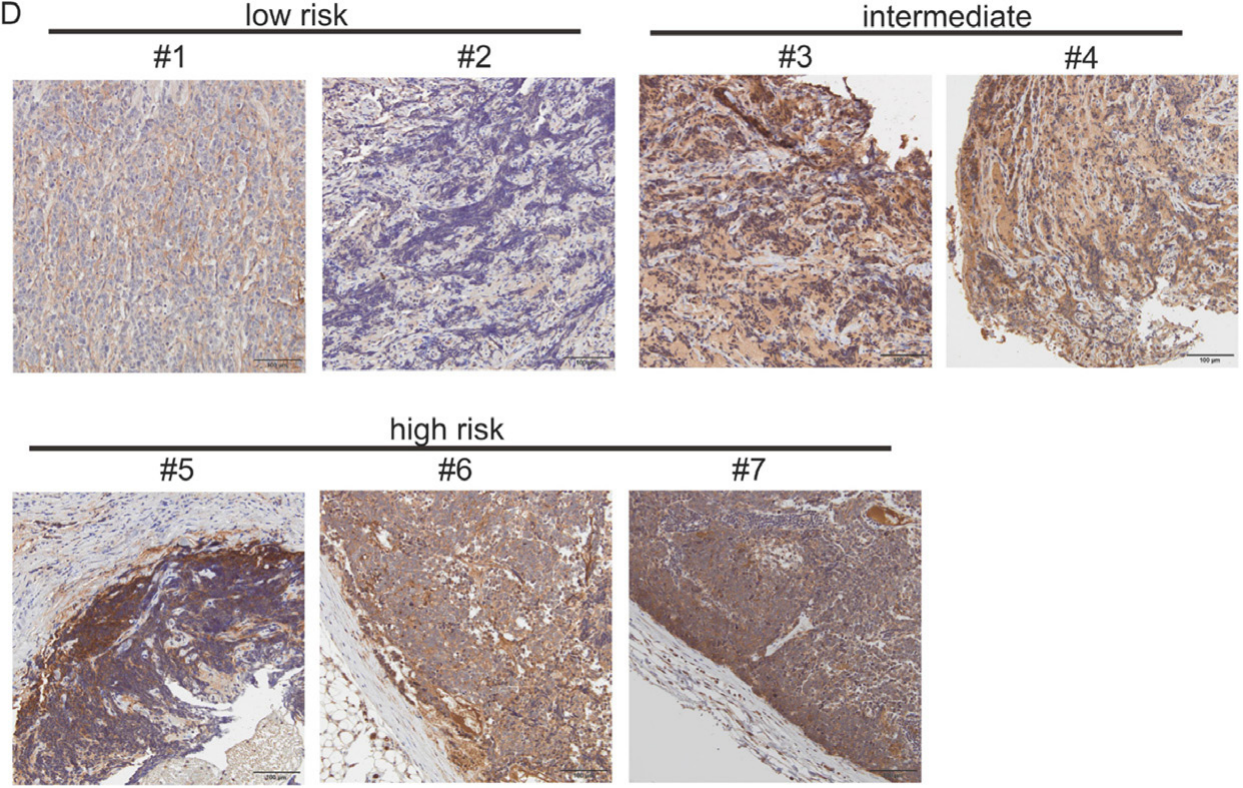
A higher expression of NCAN is closely associated with unfavorable outcomes of NB patients (**A**) Kaplan-Meier overall survival curves based on the mRNA expression level of NCAN and other CSPGs in NB patients using scan as cut-off modus. High- and low-expression groups are indicated. The *p*-values were corrected for multiple testing (Bonferroni correction). n.s.: not significant. (**B**) NCAN mRNA expression levels in various cancer cell lines. (**C**) Box plot with circles indicating NCAN expression for individuals according to MYCN status, risk and INSS classifications in the Tumor NB public public-SEQC/RPM-498 dataset from R2 (http://r2.amc.nl). (**D**) IHC staining of NCAN in tumors of clinical patients at different stages of NB. Scale bar: 100 μm. Patients’ information was shown in Supplementary Table 1.

We first investigated the mRNA expression of CSPGs associated with overall survival in NB patients, based on the public-SEQC/RPM-498 dataset in R2 (http://r2.amc.nl). In terms of other CSPGs, such as syndecans and glypicans, because it was reported that their expressions were low in both human cell lines and clinical samples of NB [10], we didn’t investigate them here. A Kaplan-Meier survival curve analysis using scan as cut-off modus indicated that a high expression of both NCAN and VCAN were closely correlated with poor prognosis (Figure 1A). In contrast, the high expression of CSPG5, CD44 and PTPRZ1 showed the significant correlations with favorable prognosis. Next, according to the mRNA expression level in various cancer types derived from the Cancer Cell Line Encyclopedia [13], the high mRNA expression of NCAN was almost unique to NB compared to other cancer cell types (Figure 1B), whereas the expression of VCAN was not specific nor the highest in NB (Supplementary Figure 1). These results suggest the involvement of NCAN in the tumorigenesis and malignancy of NB.

Consistently, the NCAN expression was significantly higher in the mycn-amplified group compared to the non-amplified group, in the high-risk group compared to the non-high-risk group, and in the stage 4 group compared to the other stages (Figure 1C). Interestingly, the patients classified as stage 4S, in which the metastasized tumors tend to spontaneously regress, showed low NCAN levels that were comparable to the NCAN levels of lower stages. In order to confirm the clinical data in public database, we performed immunostaining of several clinical sections with anti-NCAN antibody. As shown in Figure 1D, although NCAN was expressed in all clinical samples in different risk group, its staining tend to be weaker in lower risk, and stronger in higher risk patients. These results suggest that a high level of NCAN mRNA could serve as an unfavorable prognostic marker of NB.

### NCAN expression was highly upregulated in the NB tumors of TH-*MYCN* mice

Because several data from our clinical samples or cell lines indicated a relationship between NB pathogenesis and NCAN expression, we further investigated the NCAN mRNA expression profile during the tumorigenesis of TH*-MYCN* mice, an animal model in which NB tumors spontaneously develop from superior mesenteric ganglia (SMG) [14–17]. We examined the mRNA expression of CSPGs in the SMG of 2-week-old wild-type (WT) mice, the SMG of 2-week-old TH*-MYCN* homozygous mice (initial tumors), and the terminal tumors developed in homozygous or hemizygous mice based on our previous microarray dataset (GSE 43419). The TH*-MYCN* homozygous mice showed a more severe phenotype compared to the hemizygous mice in terms of tumor incidence (100% and 70%–80%, respectively).

**Figure 2:**
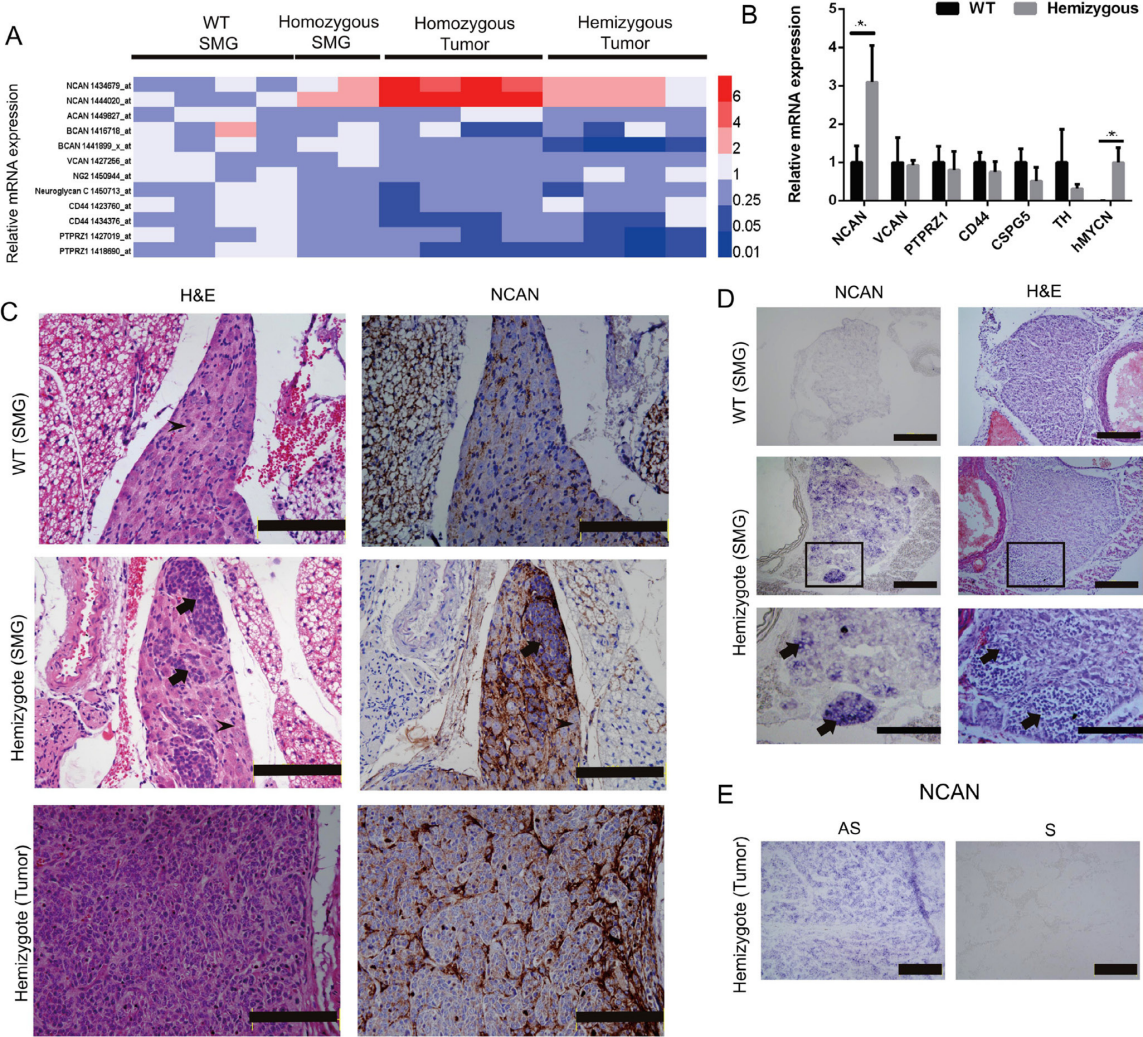
Expression of NCAN in the SMG and tumors of TH*-MYCN* mice (**A**) Heatmap of the relative mRNA expression levels of CSPGs based on the microarray analyses of TH*-MYCN* mice. (**B**) The relative mRNA expressions of NCAN, VCAN, PTPRZ1, CD44, CSPG5, TH and human MYCN in the SMG of 2-week-old WT mice and TH*-MYCN* hemizygous mice as determined by qPCR (*n* = 5). ^*^p < 0.01. (**C**) Hematoxylin and eosin (H&E) and IHC staining of NCAN in an SMG and tumor of a TH*-MYCN* mouse. Normal ganglion cells show large and slightly stained nuclei (*arrowheads*). Tumorigenic neuroblasts show small, round and deeply stained nuclei (*arrows*). Scale bar: 500 μm. (**D**) *In situ* hybridization of NCAN in the SMG of 2-week-old WT and TH*-MYCN* hemizygous mice. The tumorigenic neuroblasts were NCAN-positive (*arrows*). The boxed regions in the middle panels are magnified in the lower panels. Scale bar: 100 μm (*upper and middle*), 50 μm (*lower*). (**E**) *In situ* hybridization of NCAN in the terminal tumor of TH*-MYCN* hemizygous mice. AS: antisense, S: sense. Scale bar: 100 μm.

We observed that only NCAN among the CSPGs was significantly increased during the progression of NB in the TH*-MYCN* mice (Figure 2A). Interestingly, the NCAN expression was much higher in the tumors from the homozygotes compared to the tumors from the hemizygotes. To test the results from the microarray analysis, we carried out an RT-qPCR to examine the expression of CSPGs including NCAN in the SMG of 2–3-week-old WT and TH*-MYCN* hemizygous mice. The results demonstrated that only NCAN was significantly increased in the hemizygotes (Figure 2B), which is consistent with the microarray results. In contrast, the expression of other CSPGs was constant between the WT and hemizygous mice. ACAN and BCAN were not detected at all (data not shown).

We next examined the expression of NCAN protein by IHC. As we and others reported [14, 18], the SMG of 2-week-old TH*-MYCN* hemizygous mice contain locally accumulated neuroblasts, which is the initial lesion of NB (Figure 2C, middle left), whereas the SMG of 2-week-old WT mice consist of totally differentiated ganglion cells (Figure 2C, upper left). The IHC staining results showed that NCAN protein was highly accumulated at the extracellular region surrounding tumorigenic neuroblasts from the hemizygotes (Figure 2C, middle right). There was a low expression of NCAN in the SMG of the WT mice (Figure 2C, upper right). NCAN maintained its high expression in the terminal tumors of the hemizygous mice (Figure 2C, lower right). These results showed that NCAN protein was highly accumulated at the extracellular matrix surrounding tumorigenic neuroblasts.

Because NCAN is a secreted protein, we next addressed whether the tumorigenic neuroblasts expressed NCAN mRNA. The results of the *in situ* hybridization clearly indicated that the tumorigenic neuroblasts observed in the SMG of 2-week-old hemizygotes were intensively positive for NCAN mRNA (Figure 2D, middle and lower left). NCAN mRNA was also ubiquitously expressed in the neuroblasts occupying the terminal tumor tissue of hemizygotes (Figure 2E). We can thus conclude that NCAN is highly expressed and secreted from NB tumor cells all through the tumorigenic process.

### The exogenous expression of NCAN in NB cells induces sphere formation and potentiates malignancy

**Figure 3:**
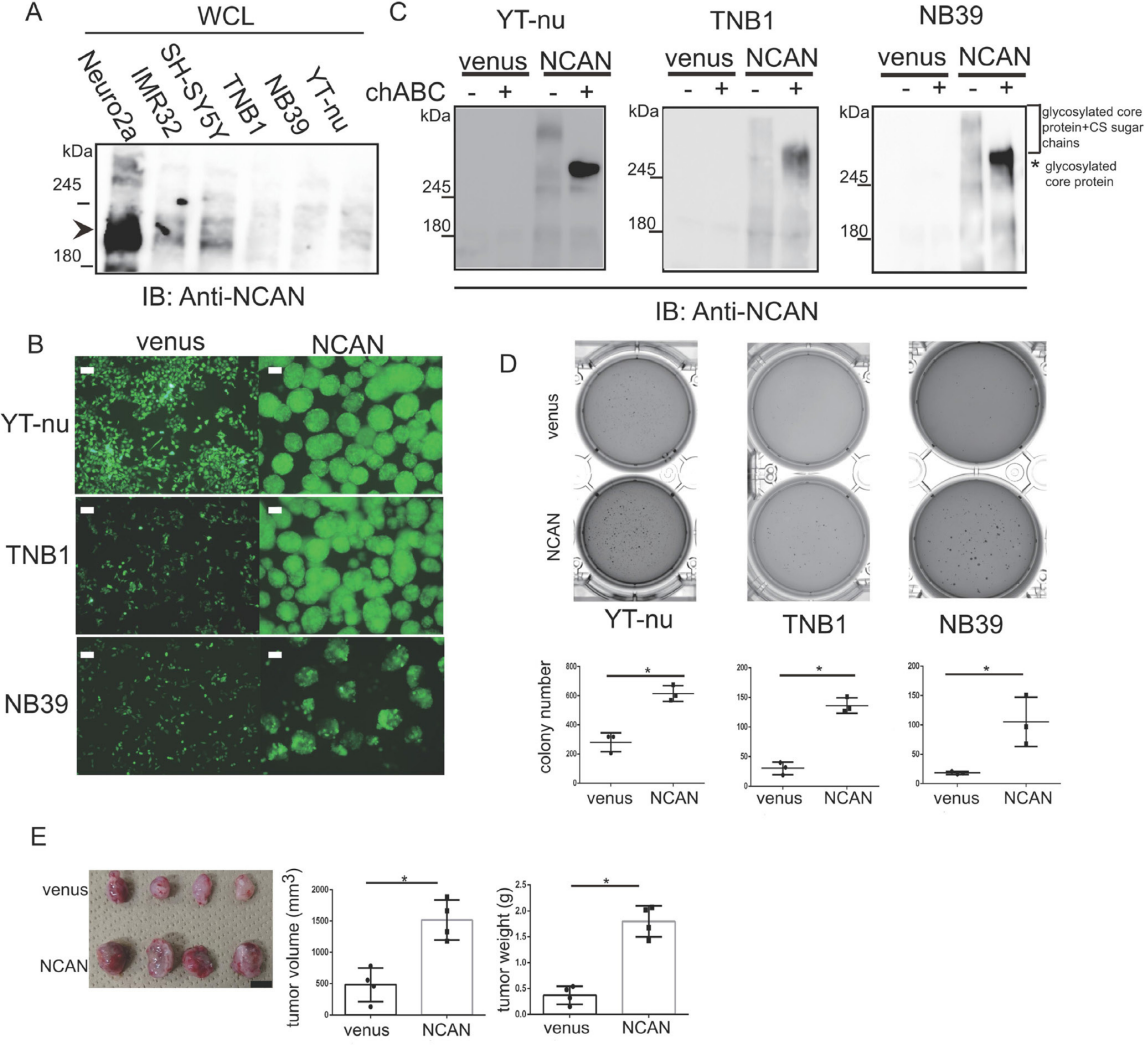
Exogenous NCAN expression in human NB cell lines induces sphere formation and potentiates malignancy **(A)** Western blot for NCAN expression in mouse (Neuro2a) and human NB cell lines. Endogenous full-length NCAN is indicated by the arrowhead. (**B)** Representative fluorescent images of venus-expressing adherent cells and NCAN-expressing sphere cells derived from YT-nu, TNB1 and NB39 cells. The pictures were taken 72 hr after infection with lentivirus overexpressing either venus or NCAN. Scale bars: 100 μm. (**C)** Western blot for NCAN expressed and secreted from the cells shown in panel B. The mediums were harvested and then untreated or treated with chABC at 37°C for 1 hr. (**D)** The anchorage-independent colony formation assay. Representative crystal violet-staining images and the colony numbers (dia. >200 μm) in soft agar are shown (*n* = 3). ^*^p < 0.001. (**E)** The subcutaneous xenografts in KSN/Slc nude mice. The image of tumors derived from either venus- or NCAN-expressing NB39 cells (*left*), quantified tumor volumes (*middle*), and weights (*right*). Tumors were dissected at 4 weeks after inoculation. ^*^p < 0.001. Scale bars: 1 cm.

We next exogenously expressed NCAN in the TNB1, NB39 and YT-nu NB cells, in which the endogenous NCAN protein was almost absent (Figure 3A), and we examined the cells’ phenotypes. Surprisingly, all three cell lines changed their morphologies into floating spheres (Figure 3B). We confirmed the expression of NCAN and secretion into the medium. The smear bands (far over 245 kDa) were detected in every medium harvested from each cell (Figure 3C). After the treatment with chondroitinase ABC (chABC) to digest CS sugar chain, those smear bands turned into approx. 270-kDa bands presumably representing the naked core protein (Figure 3C). Consistent with the report suggesting that the long CS sugar chains can mask the antigen recognized by anti-NCAN antibody [12], the chABC treatment increased the amount of responsive NCAN protein to antibody (Figure 3C). These results indicate that the extracellular NCAN was actually modified by CS.

Because tumor sphere cells in gliomas, breast cancer and colon cancer showed increased stemness and malignancy [19–21], we investigated the phenotype of those NCAN-expressing tumor sphere cells. We observed that the NCAN-expressing tumor sphere cells derived from three cell lines showed significantly greater anchorage-independent colony formation ability compared to the venus-expressing cells (Figure 3D). In addition, the NCAN-expressing tumor sphere cells exhibited a potentiated tumor-forming ability *in vivo* when they were subcutaneously inoculated into nude mice (Figure 3E). Furthermore, we found that NCAN-induced sphere cells were more resistant to the major anticancer drug in NB therapy, cisplatin, in soft agar assays (Supplementary Figure 2). These results clearly showed that the NCAN-induced tumor sphere formation was concomitant with the promotion of malignancy both *in vitro* and *in vivo*.

### Both the sugar chains and core protein of NCAN are essential for inducing sphere formation

**Figure 4:**
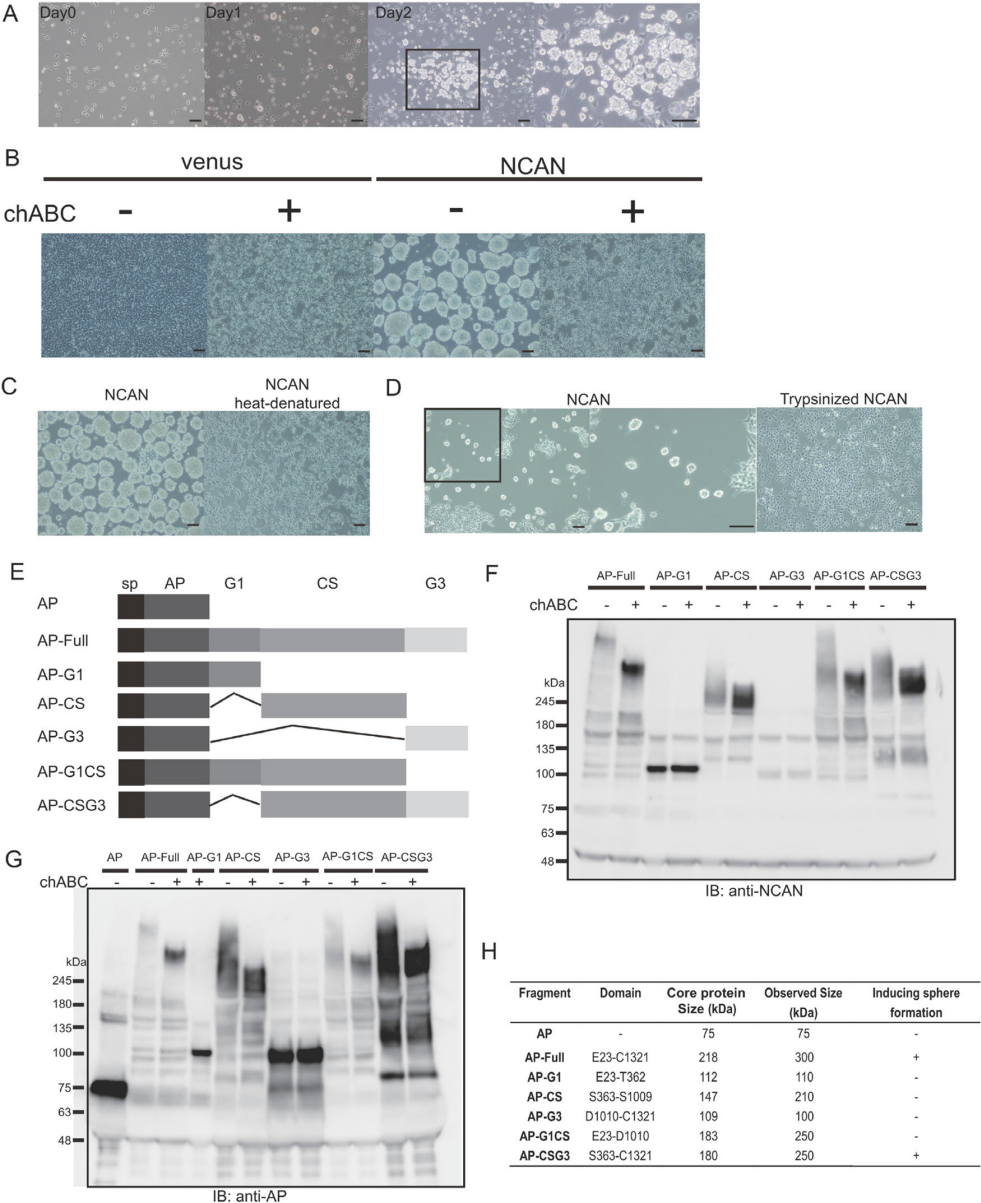
Both the CS sugar chains and core protein of NCAN are essential for sphere formation **(A)** TNB1 cells were cultured for 2 days in serum-free medium containing 10 μg/ml of recombinant NCAN. The boxed region is magnified at the right. Scale bars: 100 μm. (**B)** The treatment of TNB1 cells with conditioned medium containing overexpressed and secreted NCAN from YT-nu cells. The conditioned medium of venus-expressing YT-nu cells was prepared as a control. The conditioned media were either untreated or treated with chABC. Representative images of TNB1 cells treated with each conditioned medium are shown. Scale bars: 100 μm. (**C)** TNB1 cells were treated with conditioned medium whose NCAN core protein was heat-denatured (boiled for 15 min). Representative pictures (*left:* control, *right:* heat-denatured) are shown. Scale bars: 100 μm. (**D)** TNB1 cells were treated with conditioned medium whose proteins were digested by trypsin. The conditioned media were digested by 0.01% of trypsin (37°C for 15 min), followed by the addition of FBS to inactivate trypsin. Representative pictures (*left:* control, *right:* trypsinized) are shown. The boxed region is magnified in the middle panel. Scale bars: 100 μm. (**E)** Schematic of full-length NCAN and five other truncated versions of NCAN. NCAN consists of G1, CS and G3 domains. Every version of NCAN is fused with AP at the N-terminal. sp: signal peptide. (**F)** Western blot of each conditioned medium with anti-NCAN antibody. The media were treated or untreated with chABC. (**G)** Western blot of each conditioned medium with anti-AP antibody. The media were treated or untreated with chABC. (**H)** A series of AP-fused NCAN-containing conditioned media were examined for their ability to induce the sphere formation of TNB1 cells.

To further test the NCAN-induced tumor sphere formation, we treated TNB1 cells with human recombinant NCAN protein and then examined the phenotype. Recombinant NCAN also induced the sphere formation of TNB1 cells, indicating that NCAN was truly responsible for the sphere formation (Figure 4A). In addition, we treated TNB1 cells with NCAN-containing conditioned medium harvested from NCAN-overexpressing YT-nu cells. The results revealed that the conditioned medium also could induce sphere formation (Figure 4B).

Because NCAN consists of core protein and CS sugar chains, we next addressed the contribution of those components to tumor sphere formation. When TNB1 cells were co-treated with NCAN-containing conditioned medium and chABC to digest CS, the sphere formation was completely abolished (Figure 4B). When TNB1 cells were treated with either heat-denatured or pre-trypsinized NCAN-containing conditioned medium in which the NCAN core protein is impaired, the sphere formation was also abolished (Figure 4C, 4D). Taken together, these findings led us to conclude that both the CS sugar chains and the core protein of NCAN were necessary for the induction of tumor sphere formation.

We next focused on the domain structure of the NCAN core protein. As reported [22], NCAN protein is roughly divided into three domains: the N-terminal G1 domain, the central CS domain, and the C-terminal G3 domain. To investigate which domain(s) are involved in sphere formation, we constructed a series of truncated versions of NCAN (Figure 4E). Alkaline phosphatase (AP) was fused at the N-terminus of every construct to serve as an epitope tag. First, we confirmed the expression and secretion of these truncated NCANs. We immunoblotted with anti-NCAN (Figure 4F) or anti-AP (Figure 4G) antibodies, because AP-G3 construct could not be detected by anti-NCAN antibody. It was notable that the constructs containing the CS domain showed smear bands in the untreated condition, and they showed lower and stronger bands in the chABC-treated condition (Figure 4F, 4G). These results suggested that the CS sugar chains were mainly attached on the CS domain.

We then treated TNB1 cells with the conditioned medium containing the NCANs, and we examined the tumor sphere formation. As summarized in Figure 4H and Supplementary Figure 3, only AP-Full and AP-CSG3 could successfully induce tumor sphere formation. Interestingly, AP-CS was not functional, whereas AP-CS seemed to be glycosylated (Figure 4F, 4G). The G3 domain can be expected to possess some function. We concluded that the CS and G3 domains are essential for the NCAN core protein.

### NCAN stimulates NB cells to inhibit growth arrest and to potentiate undifferentiated state

**Figure 5:**
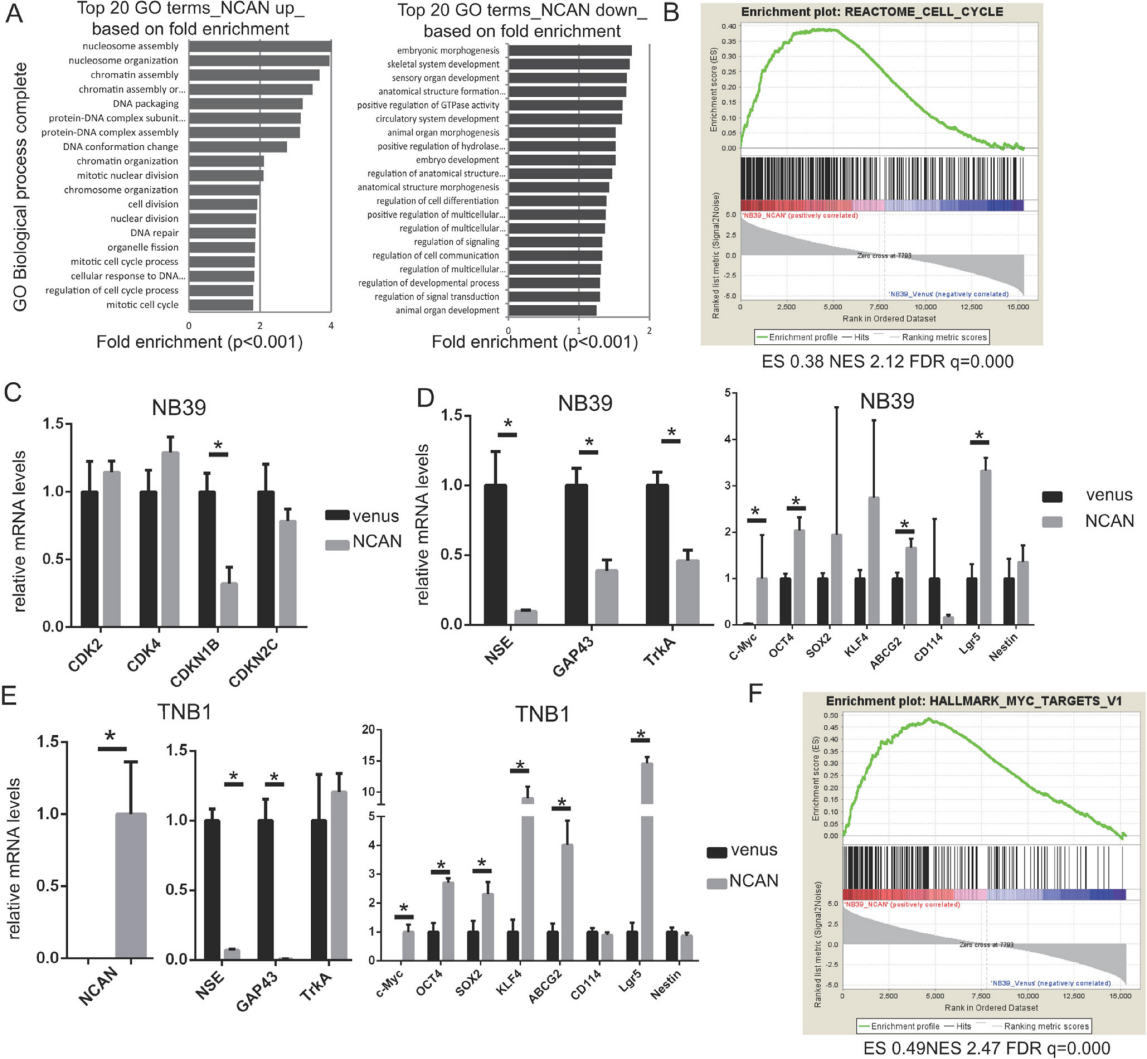
NCAN potentiates NB malignancy by increasing cell division and dedifferentiation **(A)** Results of the GO analysis of upregulated (*left*) or downregulated genes (*right*) in NB39 cells treated with NCAN-containing conditioned medium compared with those treated with venus-containing conditioned medium. The top 20 GO biological process terms based on fold enrichment are shown. (**B)** The GSEA results indicated that the gene set related to the cell cycle was significantly enriched in NB39 cells treated with NCAN-containing conditioned medium. (**C)** The RT-qPCR results of cyclin-dependent kinases (CDKs) and CDK inhibitors in NB39 cells treated with NCAN-containing conditioned medium compared with those treated with venus-containing conditioned medium. ^*^p < 0.01. (**D)** The results of the RT-qPCR of differentiation and stemness marker genes in NB39 cells treated with NCAN-containing conditioned medium compared with those treated with venus-containing conditioned medium. ^*^p < 0.01. (**E)** The RT-qPCR of the differentiation and stemness marker genes in TNB1 cells infected with NCAN-expressing or venus-expressing lentivirus. ^*^p < 0.01. (**F)** The GSEA indicated that the set of MYC targets was significantly enriched in NB39 cells treated with NCAN-containing conditioned medium.

To clarify the mechanisms underlying the induction of tumor sphere formation by NCAN and the potentiation of the malignancy of NB cells by NCAN, we investigated the comprehensive mRNA expression pattern by performing a DNA microarray analysis. NB39 cells were treated with conditioned medium containing NCAN or with control medium, and their RNA samples were subjected to the DNA microarray analysis. As a result, we identified 1,811 upregulated (>2-fold, *p* < 0.05) genes and 2,601 downregulated (<0.5-fold, *p* < 0.05) genes in NCAN-treated NB39 cells compared to the controls. Among the 1,811 upregulated genes, the Gene Ontologies (GO) for nucleosome assembly, chromatin assembly, cell cycle and cell division were enriched (Figure 5A). Consistently, a gene set enrichment analysis (GSEA) revealed that the cell-cycle gene set was significantly enriched (Figure 5B).

To test these results of the DNA microarray, we carried out a RT-qPCR, and we observed that the cell-cycle inhibitor *CDKN1B* was significantly downregulated in NCAN-treated NB39 cells (Figure 5C). In terms of the downregulated genes in NCAN-treated NB39 cells, the GOs for morphogenesis, development and cell differentiation were enriched (Figure 5A). Based on these results, we speculate that the NCAN-treated cells were less differentiated than control ones. Consistent with this notion, the RT-qPCR revealed that some neuronal differentiation markers, such as *NSE* and *GAP43*, were significantly downregulated in NCAN-treated NB39 cells (Figure 5D) and NCAN-overexpressing TNB1 cells (Figure 5E). *TrkA* was downregulated only in NB39 cells (Figure 5D, 5E).

In contrast, putative stemness markers such as *c-Myc*, *OCT4*, *SOX2*, *KLF4, ABCG2* and *Lgr5* were upregulated in the NCAN-stimulated cells (Figure 5D, 5E). The MYC target gene set in GSEA was also significantly enriched (Figure 5F). Taken together, these results suggest that the NCAN-treated cells are dedifferentiated. Collectively, our results indicate that NCAN potentiates the malignancy of NB cells by promoting cell division and maintaining an undifferentiated status.

### NCAN is essential for the growth of tumor sphere cells derived from TH-*MYCN* mice both *in vitro* and *in vivo*

**Figure 6:**
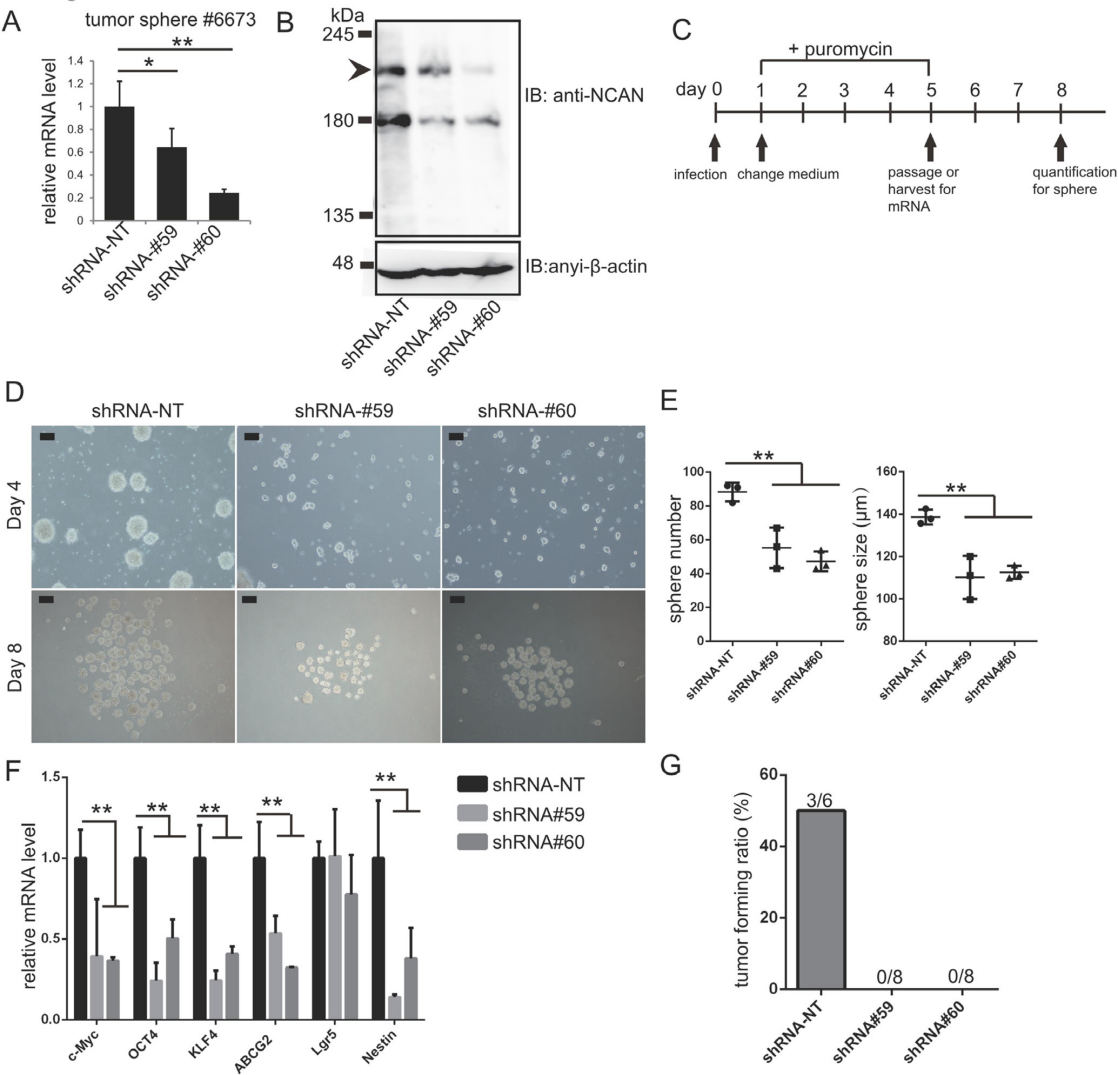
NCAN is essential for the malignancy of tumor spheres derived from TH-*MYCN* mice (**A**) RT-qPCR revealed the efficient knockdown of NCAN mRNA by two independent shRNAs in the tumor sphere line #6673 derived from TH*-MYCN* mice. ^*^p < 0.05, ^**^p < 0.01. (**B**) Western blot for mouse endogenous NCAN showing the efficient knockdown by shRNAs in the tumor sphere line #6673 derived from TH*-MYCN* mice. (**C**) Schematic of NCAN knockdown in mouse tumor sphere cells. (**D**) Representative images of tumor spheres after the knockdown of NCAN. Day 4: Four days after infection with shRNA-expressing lentivirus. Day 8: Just before the quantification of spheres. Scale bars: 100 μm. (**E**) Quantification of the sphere numbers and sizes shown in panel C. ^**^p < 0.01. (**F**) RT-qPCR analysis of stemness marker genes in each tumor sphere. ^**^p < 0.01. **(G**) Here, 1 × 10^5^ tumor sphere cells expressing each shRNA were subcutaneously inoculated into KSN/Slc nude mice. The tumor forming ratio is indicated (*n* = 6 for shRNA-NT, *n* = 8 for shRNA#59 and shRNA#60).

Lastly, we addressed the involvement of NCAN in the formation of tumor spheres derived from TH*-MYCN* mice. We previously established the tumor sphere lines cultured from terminal tumor tissues of TH*-MYCN* mice [14, 23]. Here we confirmed that mouse NCAN was successfully knocked down with two independent shRNA (Figure 6A, 6B). We evaluated the tumor sphere formation and gene expression as indicated by the scheme in Figure 6C. The knockdown of NCAN resulted in reductions of both the sphere number and size (Figure 6D, 6E). The putative stemness marker genes, such as *c-Myc, OCT4, KLF4, ABCG2* and *Nestin*, were suppressed in these cells (Figure 6F). These results were consistent with our conclusion obtained from NB cell lines that NCAN maintained the undifferentiated status (Figure 5). On the other hand, we also found that the knockdown of NCAN in adherent NB cell lines also resulted in growth suppression (Supplementary Figure 4).

To further evaluate the involvement of NCAN in mouse tumor sphere cells, we carried out allografts in which the mouse tumor sphere cells were subcutaneously inoculated into the same strain of wild-type mice. As a result, the control tumor sphere cells developed subcutaneous tumors at a certain frequency, whereas the NCAN-knocked down cells could not form subcutaneous tumors at all (Figure 6G, Supplementary Figure 5). These data from TH*-MYCN* mice-derived tumor sphere cells strongly supported the essential involvement of NCAN on tumor sphere cells.

## DISCUSSION

In this study, we identified NCAN as an unfavorable prognostic marker in the public clinical database R2 (Figure 1). The immunohistochemistry analysis of the TH*-MYCN* mice revealed the accumulation of NCAN at the extracellular region surrounding tumorigenic neuroblasts, which suggests the involvement of NCAN in the tumorigenesis of NB (Figure 2). We found that exogenous NCAN can induce the sphere formation of NB cells, which showed the more malignant phenotype (Figures 3, 4). The tumor sphere formation was concomitant with the particular gene expression profile implicating promoted cell division and undifferentiated state (Figures 5, 6). Taken together, these results suggest that NCAN is involved in the tumorigenesis and malignancy of NB.

Previous reports mentioned that the mRNA of NCAN was detected in neoplastic mammary glands in mice [24], and that it was significantly increased in several cancer types [25, 26]. These findings implied that NCAN might be generally involved in tumor pathogenesis. However, our present study appears to be the first to address the actual function of NCAN in cancer.

Because NCAN induced both tumor sphere formation and the expression of stemness-related genes, our findings suggest that NCAN may function as a component of the extracellular matrix, which comprises the particular environment for stem cells, i.e., the niche. There are several studies whose results are consistent with our hypothesis. For example, neural precursor cells cultured as neurospheres secreted lectican family CSPGs including NCAN, and their expression was attenuated as differentiation proceeded [27]. Moreover, CSPGs including NCAN were detected in the milieu of neural stem cells, and the CSPGs regulated the cells’ proliferation as microenvironmental factors [28]. Taking these reports into consideration, it is conceivable that NCAN is involved in the dedifferentiation property of NB cells.

The next subject to be addressed is the molecular mechanism of NCAN signaling. The important NCAN receptor might be PTPRσ. The glycosaminoglycan chains of both CSPGs and HSPGs can be recognized by the immunoglobulin-like domain of PTPRσ. Importantly, PTPRσ bound to CSPGs is monomerized, which results in its activation to dephosphorylate downstream substrates including receptor tyrosine kinases. On the other hand, PTPRσ bound to HSPGs is oligomerized, which results in its inactivation [8, 29]. Thus, CSPGs and HSPGs play opposite roles in neural regeneration and neuronal extension.

Interestingly, it was reported that both neural stem cells and their progeny express PTPRσ, and that the neurons differentiated from PTPRσ-deficient neurospheres showed enhanced neurite outgrowth, which was the typical phenotype of neural differentiation [30]. These results are consistent with the hypothesis that NCAN bind to and activate PTPRσ, and that this axis maintains the undifferentiated status of neurons.

In NB, HSPGs help heparin-binding epidermal growth factor-like growth factor (HB-EGF) to bind to EGFR, which results in the differentiation of NB cells via ERK1/2 and STAT3 [31]. HSPGs, such as glypicans and syndecans, were also shown to induce the differentiation of NB cells via FGF2-FGFR1 signaling [9, 10]. They are located on the cell membrane, and are shed by matrix metalloproteinases. The released extracellular domains function as co-receptors. Importantly, NCAN, as a component of extracellular matrix, is reported to bind to HSPGs including glypican-1 and syndecan-3 [32] and FGF2 [33] via its C-terminal domain, which was shown to be essential for tumor sphere formation in our experiments (Figure 4E–4H). This might result in the sequestration of those HSPGs from FGFR1, which could suppress the differentiation of NB cells. Alternatively, the NCAN-PTPRσ axis-mediated inactivation of EGFR or FGFR1 might also be possible. As described above, PTPRσ can dephosphorylate and inactivate receptor tyrosine kinases such as EGFR and FGFR1. The NCAN-PTPRσ axis could antagonize those HSPG-mediated differentiating signals by dephosphorylating EGFR and FGFR1. In summary, we have identified NCAN as a novel and clinically relevant malignant factor of NB. NCAN could be a potent therapeutic target to kill the malignant cells.

## MATERIALS AND METHODS

### Cell culture and reagents

The human NB cell lines IMR32, NB39 and TNB1 were obtained from RIKEN Cell Bank (Tokyo). The cell line YT-nu was obtained from the Carcinogenesis Division, National Cancer Center Research Institute (Tokyo). SH-SY5Y cells and the mouse NB cell line Neuro2a were obtained from American Type Culture Collection (ATCC) (Manassas, VA, USA). HEK293T cells were obtained from IFOM (Milan, Italy).

IMR32 and Neuro2a were cultured in minimum essential medium (MEM) with 10% FBS and 1% non-essential amino acid solution (NEAA) (Sigma, St. Louis, MO). NB39, SH-SY5Y and HEK293T cells were cultured in Dulbecco’s Modified Eagle Medium (DMEM) with 10% FBS. TNB1 and YT-nu cells were cultured in RPMI-1640 medium with 10% FBS. All cells were maintained in incubators at 37°C with 5% CO_2_. In the beginning of each experiment, adequate numbers of frozen cell stocks were prepared. Every 2 to 3 months, we cultured new stocks in order to maintain the stocks’ original condition. The viability, growth rate and morphology of the cells were examined by light microscopy. Chondroitinase ABC (chABC) and human recombinant NCAN were purchased from R&D Systems (Minneapolis, MN).

### Conditions of sphere culture

To prepare the conditioned medium containing NCAN, we seeded 3 × 10^6^ YT-nu cells stably infected with the NCAN-expressing lentivirus on 10-cm dishes. For the AP-fused full-length NCAN and other truncated versions, 3 × 10^6^ HEK293T cells stably transfected with the expression plasmids were seeded on 10-cm dishes, and 24 hr later, the medium was replaced with RPMI-1640. After an additional 2 days, the medium was harvested and centrifuged at 3,000 rpm for 5 min. The supernatant was aliquoted and stored at −80°C. For the conditioned medium treatment, 1 × 10^5^ TNB1 cells were seeded on 6-well plates, and 24 hr later, the cells were washed with phosphate-buffered saline (PBS) and treated with conditioned medium for 2 days. The floating sphere cells were then collected and centrifuged at 3,000 rpm for 5 min. The cells were resuspended in fresh RPMI-1640 medium and cultures for an additional 3–4 days.

For the recombinant NCAN treatment, 1 × 10^5^ TNB1 cells were seeded on 6-well plates, and 24 hr later, they were washed with PBS and treated with recombinant NCAN (10 μg/ml) in serum-free RPMI-1640 medium for 2 days.

NCAN-expressing sphere cells and conditioned medium-treated sphere cells were cultured in tissue culture dishes with medium containing 10% FBS. Recombinant NCAN-treated sphere cells were cultured in tissue culture dishes with serum-free medium.

The established NB sphere cells from terminal tumors of TH*-MYCN* mice [15] were cultured in DMEM/Ham’s F12 medium supplemented with 15% FBS, 1% NEAA, 1% sodium pyruvate (Sigma), 10 ng/ml EGF, 15 ng/ml FGF (PeproTech, Rocky Hill, NJ), 2% B27 (the human leukocyte antigen) and 55 μM β-mercaptoethanol (Invitrogen, Carlsbad, CA) [23]. The mouse sphere cells were cultured in non-treated dishes.

The spheres were pictured by UPLSAP040X microscope, and analyzed by cellSens standard software (Olympus, Japan). The colonies whose diameter was more than 40 μm were counted.

### Plasmids

Full-length and truncated versions of human NCAN were cloned from human brain cDNA (Clontech, Tokyo), and inserted into the XhoI-XbaI site of the vector pAPtag-5 (GenHunter, Nashville, TN). The signal peptide cloned from SH-SY5Y cells and alkaline phosphatase (AP)-NCAN fusion cDNA cut out from pAPtag-5 were then inserted into the NheI-BamHI site of CSII-CMV-MCS-IRES2-venus (RIKEN BioResource Center, Tokyo), which is a lentivirus-based expression vector. The primer sequences are shown in Supplementary Table 2.

### Animals

The TH*-MYCN* mice were maintained with standard chow and water in an animal facility under a controlled environment. The mice were crossed with 129^+Ter^/SvJcl wild-type (WT) mice. Superior mesenteric ganglion (SMG) tissues were dissected from 2–3-week-old WT and TH*-MYCN* hemizygous mice. Sphere cells were cultured from the tumors developed in 3-month-old hemizygous mice. Four-week-old WT mice were used as the recipients of allografts. All animal experiments were approved by the Animal Care and Use Committee of Nagoya University Graduate School of Medicine (Nagoya, Japan).

### Reverse transcription and real-time quantitative PCR

The whole RNA extraction from SMG and cultured cells and the reverse transcription (RT) were carried out as described [17]. RT was carried out with ReverTraAce (Toyobo, Tokyo). The quantitative polymerase chain reaction (qPCR) was carried out with THUNDERBIRD SYBR qPCR Mix (Toyobo) and an Mx3000P or Mx3005P Real-time QPCR system (Agilent Technologies, Santa Clara, CA). The relative expression levels were calculated according to the manufacturer’s instructions (ΔΔCt method). The primer sequences are shown in Supplementary Table 2.

### Immunohistochemistry

Dissected tissues were fixed in 4% paraformaldehyde, dehydrated, and embedded in paraffin blocks according to the standard methods. Sections with a thickness of 5 μm were subjected to heat retrieval and then incubated with anti-NCAN polyclonal antibody (R&D Systems) at a concentration of 1:200 in 5% bovine serum albumin overnight at 4°C. The sections were next incubated with biotin-conjugated donkey anti-sheep IgG antibody (1:100, Jackson ImmunoResearch, West Grove, PA) for 1 hr at room temperature. The signals were visualized with a VECTASTAIN Elite ABC Standard Kit (Vector Laboratories, Burlingame, CA) and 3, 3′-diaminobenzidine (DAB, Dako, Glostrup, Denmark).

### *In situ* hybridization

For the detection of NCAN mRNA, we used *in situ* hybridization. Two independent probes were cloned (template: Neuro2a cDNA, DNA polymerase: KOD FX (Toyobo) into the EcoRI-BamHI site of pBluescript II SK (+) (Stratagene, San Diego, CA). Antisense and sense probes were transcribed and labeled with a DIG RNA Labeling Kit (Roche, Basel, Switzerland). After purification, the probes were kept at −80°C. Continuous 5 μm frozen sections were hybridized with either antisense or sense probe, and visualized according to the standard protocol supplied by Roche. The primer sequences for each probe are shown in Supplementary Table 2.

### Western blotting

The media were harvested and digested by chABC (1:100, R&D Systems) for 1 hr at 37°C. After the separation with 6% or 10% sodium dodecyl sulfate-polyacrylamide gel electrophoresis (SDS-PAGE) gels or 5%–20% gradient gels, the proteins were transferred onto nitrocellulose membranes. They were incubated with anti-NCAN antibody (1:1000, R&D Systems), anti-alkaline phosphatase (AP, 1:1000, R&D Systems) or anti-β-actin antibody (1:10000, Sigma), respectively. For the detection of secondary antibodies, i.e., horseradish peroxidase (HRP)-conjugated donkey anti-sheep IgG (1:5000, R&D Systems) and goat anti-mouse IgG (1:10000, Jackson ImmunoResearch), the membranes were analyzed with an Amersham Imager 600 (GE Healthcare, Little Chalfont, UK).

### Packaging of lentivirus

Non-targeting shRNA and anti-mNcan shRNA were purchased from Sigma. The plasmid encoding either shRNA or NCAN was cotransfected with the lentivirus packaging plasmids pMD2.G (plasmid 12259, Addgene, Cambridge, MA) and psPAX2 (plasmid 12260, Addgene) into HEK293T cells. The titer of virus was determined with the use of a QuickTiter Lentivirus Titer kit (CellBiolabs, San Diego, CA). The shRNA sequences were as follows.

Mouse NCAN shRNA#59: 5′-ccggctagtaatgtgacgatgaatcctcgaggattcatcgtcacattactagtttttg-3′

Mouse NCAN shRNA#60: 5′-ccggtatgcagcccttgcgagaatgctcgagcattctcgcaagggctgcatatttttg-3′

Mouse NCAN shRNA#61: 5′-ccggcaggcgtcgtgttccattatcctcgaggataatggaacacgacgcctgtttttg-3′

Human NCAN shRNA#55: 5′-ccgggagaaccagccggacaatttcctcgaggaaattgtccggctggttctcttttttg-3′

Human NCAN shRNA#58: 5′- ccgggccaatagagttgaggcacatctcgagatgtgcctcaactctattggctttttg-3′

### Infection and transfection

First, 1 × 10^5^ NB cells were infected with 1 ml of expression virus in the presence of 8 μg/ml polybrene (Sigma). In addition, 1 × 10^4^ mice tumor sphere cells were infected with 500 μl of shRNA virus in the presence of 8 μg/ml polybrene. Puromycin at the final concentration of 1 μg/ml was added to select the cells infected with shRNA-expressing virus, followed by the establishment of cells stably expressing shRNA. For transfection, 1 × 10^5^ of HEK293T cells were transfected with 4.5 μg of plasmid and 18 μl of FUGENE HD (Promega, Madison, WI).

### Monolayer proliferation assay

Firstly, 1 × 10^5^ of NB cells were seeded into 6-well plates. Twenty-four hours after infection, the virus was removed, and the cells were washed for 3 times with PBS, followed by the addition of fresh medium. After 72 hours, the cells were washed for 3 times with PBS. Then 2 ml of serum-free medium with 100 μM resazurin was added. After 2 hours, 100 μl of medium was transferred into 96-wells plate, and the fluorescence was measured by POWERSCAN4 (Bio Tek, λexc = 560 nm, λem = 590 nm).

### Soft agar assay

For the soft agar assay, 1 ml of bottom agar (0.5% agar/RPMI or DMEM + 10% FBS) and 1 ml of top agar (0.33% agar/RPMI or DMEM + 10% FBS) were plated on 6-well plates (*n* = 3) with or without cisplatin (0.125 μM, 0.5 μM, 2 μM, and 8 μΜ, respectively). Negative control or NCAN-overexpressing cells (4,000 YT-nu, TNB1 or NB39 cells) were seeded into the top agar. Two weeks later, the colonies were stained with crystal violet and counted with an ImageQuant LAS-4010 digital imaging system (GE Healthcare).

### Xenograft model in nude mice

NB39 cells (10^6^ negative control or NCAN-overexpressing cells in 50% Matrigel (BD Biosciences, Franklin Lakes, NJ)) were subcutaneously inoculated into the flank of 5-week-old KSN/Slc nude mice (*n* = 6; SLC Japan, Tokyo). Four weeks later, the mice were sacrificed for the measurement of the tumor weights and volumes. Volumes were calculated with the following formula: volume = (width in mm)^2^ × (length in mm)/2.

### Gene expression microarray

Expression data of TH*-MYCN* mice obtained by GeneChip Mouse Genome 430 2.0 array (Affymetrix, Santa Clara, CA) have been previously published [34], and deposited in the Gene Expression Omnibus database of NCBI under the accession number GSE43419. For the conditioned media treatment, total RNAs from cells treated with either negative control or overexpressed NCAN-containing conditioned media were extracted. Their quantity and quality were evaluated with an Agilent RNA6000 Nano Kit and Agilent 2100 Bioanalyzer (Agilent Technologies). In addition, 200 ng of RNAs was labeled with Cyanine3 with a Low Input Quick-Amp Labeling Kit (Agilent Technologies) and purified and hybridized to the SurePrint G3 Human Gene Exp 8 × 60 K Microarray (G4851C, Agilent Technologies). The scanning of microarrays and data extraction from scanned images was performed with a Sure Scan Microarray Scanner (G4900DA, Agilent Technologies). Labeling, hybridization, washing and scanning were all performed according to the manufacturer’s instructions. Scanned signals were analyzed with the visualization and data sharing tool Subio Platform (Subio, Kagoshima, Japan).

### Allografts

For the allografts, 1 × 10^5^ control or NCAN-knocked down cells mixed with 50% Matrigel were subcutaneously inoculated into the flank of 4-week-old 129^+Ter^/SvJcl mice (CLEA, Tokyo), and 3 weeks later the tumors were dissected and weighed.

### Clinical neuroblastoma samples

Written informed consent was obtained from the parents of all patients. The study was approved by the Ethics Committee of Nagoya University Graduate School of Medicine.

### Statistical analysis

Results are presented as the mean ± SD. Differences between pairs of group were evaluated by Studentʼs *t*-test.

## SUPPLEMENTARY MATERIALS FIGURES AND TABLES



## Abbreviations

ACAN: aggrecan
AP: alkaline phosphatase
BCAN: brevican
CDKN1B: cyclin-dependent kinase inhibitor 1b
chABC: chondroitinase ABC
CSPG: chondroitin sulfate proteoglycan
DSPG: dermatan sulfate proteoglycan
EGF: epidermal growth factor
FBS: fetal bovine serum
FGF2: fibroblast growth factor 2
GAP43: growth associated protein 43
GSEA: gene set enrichment analysis
HA: hyarulonic acid
HB-EGF: heparin-binding epidermal growth factor-like growth factor
HSPG: heparan sulfate proteoglycan
IHC: immunohistochemistry
KSPG: keratan sulfate proteoglycan
NB: neuroblastoma
NCAN: neurocan
NEAA: non-essential amino acid
NSE: neuron-specific enolase
PTPRσ: protein tyrosine phosphatase receptor sigma
shRNA: short hairpin RNA
SMG: superior mesenteric ganglion
SSEA-1: stage-specific embryonic antigen 1
TH: tyrosine hydroxylase
TrkA: tropomyosin receptor kinase A
VCAN: versican
WT: wild-type
