# Supplementary material for: Neurocan, an extracellular chondroitin sulfate proteoglycan, stimulates neuroblastoma cells to promote malignant phenotypes
Source: Oncotarget. 2017 Nov 15;8(63):106296–310. doi: 10.18632/oncotarget.22435 (PMC5739734; doi:10.18632/oncotarget.22435)
Supplement: Supplementary file 1 [file oncotarget-08-106296-s001.pdf]

Neurocan, an extracellular chondroitin sulfate proteoglycan, stimulates neuroblastoma cells to promote malignant phenotypes

SUPPLEMENTARY MATERIALS

Supplementary Table 1: Patients' information

Number	Sex[1]	Age[2]	Risk[3]	Stage[4]	*MYCN*[5]
#1	F	9m	low	1	NA
#2	M	0	low	1	NA
#3	M	1m	intermediate	4	NA
#4	F	1m	intermediate	3	NA
#5	F	1y	high	4	AMP
#6	F	1y	high	4	AMP
#7	F	2y	high	4	AMP

[1]sex: F, female; M, male
[2]age: y, year; m, month; 0, less than 1month
[3]Risk: according to histological checking
[4]Stage: according to INSS stage system
[5]N-myc amplification status: NA, non-amplication; AMP, amplification

Table 2: Primer information

	Forward primer sequence 5′-3′	Reverse primer sequence 5′-3′
RT-qPCR primers		
Mouse Gapdh	ggtggtgaagcaggcatctg	ggaggccatgtaggccatga
Mouse TH	gcccagttctcgcaggacat	cacgaagtagacgggctggt
Human MYCN	cgaccacaaggccctcagta	cagccttggtgttggaggag
Mouse Ncan	ctggtggtccccatggatga	ccatgcaggcaggggttgtt
Mouse Vcan	ggctctggtgatgcgcttct	ccgggatcccagtggcaaaa
Mouse Ptprz1	cccccagtgttggtgtgtca	ccctagccaccagcagagaa
Mouse CD44	ccccggaccagtgtatgaca	gcaaggcccctaggaaggaa
Mouse Cspg5	ggacctggcctcaggagaaa	gccacgcacatcacctggaa
Human CDK2	caagcctcctgctgccacaa	ggggactggggccaatcatt
Human CDK4	gagcacagctgctgctggaa	ggaccctccatagcctcaga
Human CDKN1B	ctgcgtaggggcgctttgtt	cgttctccaagtcccgggtt
Human CDKN2C	ggtggtggagttcctggtga	ggagccctccccacgtttat
Human NSE	ccccagggtgcccagaactt	cctgacgctcccatcacaga
Human GAP43	gcctgatgaggcccggcaaa	ctccttggctggggcatctt
Human TrkA	ctgggggagctgagaaacct	ggcacaccacagctggcatt
Human MYC	gcccagtgaggatatctgga	atcgcagatgaagctctggt
Human OCT4	ggtgcctgcccttctaggaa	ctgcccccacccctttgtgtt
Human SOX2	cgaactggagggggggagaaa	ccaccctccccaggttttct
Human KLF4	ggaaggagcccagccagaaa	cctcccccaactcacggatt
Human NCAN	cagccaccccagacctgttt	agggctggtctccccagttt
Mouse Myc	ctctgggctctcccatgcat	caggccaggctcctgatcaa
Mouse Oct4	ccgacgcttggcgggaaaaa	cccgctgcaatgggcaaagt
Mouse Klf4	cctggccatcggacctactt	gtcctccggagtccgaagaa
Human β-ACTIN	cctggcacccagcacaatgaa	ctaagtcatagtccgcctagaagca
Human CD133	ggctgtgcgggaactccttt	cagccccaggacacagcata
Human CD114	gaggctcggaaggtgttgca	gcccaagtcaggctgcagtt
Human ABCG2	gagggccaggagtccagtaa	ccaggagtggtcagattcct
Human Nestin	gcatgggggaggagtcgttt	agctctgccacctggctact
Human LGR5	ctgctgggtggcagcaagta	gcaggaagtgggactaccac
Mouse CD133	gcctctccctcctggtgatt	ggctgtcgccatggccttaa
Mouse CD114	ctgcctgatgggacccaaga	cgggggatggagcagttgtt
Mouse ABCG2	cctggtcctctccctgcttt	ggcctcagtgagatgcagca
Mouse nestin	gccgagcaggccactgaaaa	ggcaaggggggaagagaagga
Mouse Lgr5	ccaggccgtctgtgatcagt	cagggaaggacgacaggaga
in situ hybridization		
probe1	ggaattcgctggggatcaggacacaca	cgggatccgtagcagagcttaggcgaca
probe2	ggaattcgcttcagagagtgaccagag	cgggatccgtggtctgaaatggggtttc
plasmid		
Full length	agtcctcgaggaacagggcacacaggatatc	agtctctagatcagcagaaattcccttcgtcctt
G1 domain	agtcctcgaggaacagggcacacaggatatc	agtctctagatcacgtggggtgatgagctcggaa
CS domain	agtcctcgagtcacaacatggagacctagag	agtctctagatcatgagtgcacctcctccgcacc
G3 domain	agtcctcgaggatccctgtgagaacaaccct	agtctctagatcagcagaaattcccttcgtcctt
G1CS domain	agtcctcgaggaacagggcacacaggatatc	agtctctagatcatgagtgcacctcctccgcacc
CSG3 domain	agtcctcgagtcacaacatggagacctagag	agtctctagatcagcagaaattcccttcgtcctt
signal peptide	ctagctagcatgggggccccgtttgtctg	ccgctcgagcccagccacaaagagcagca

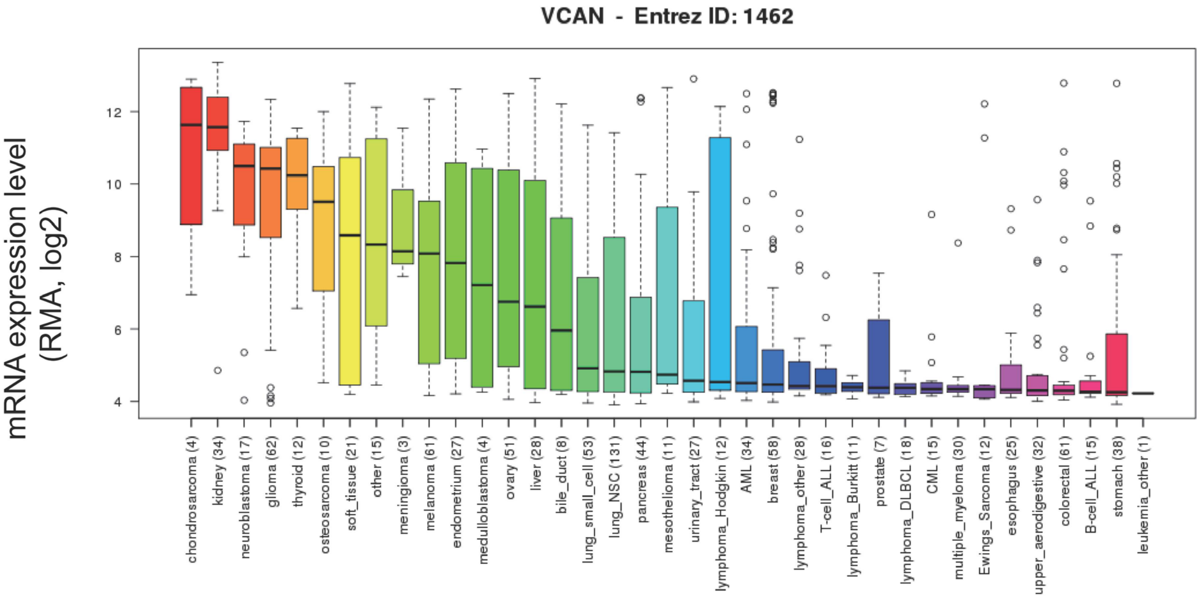

Supplementary Figure 1: VCAN mRNA expression levels in various cancer cell lines. Data was obtained from the cancer cell line encyclopedia (http://portals.broadinstitute.org/ccle/home).

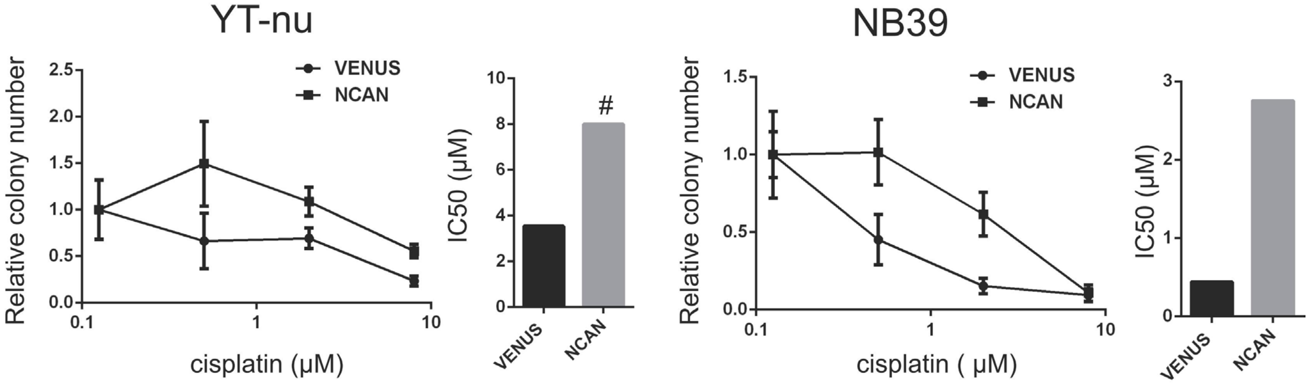

Supplementary Figure 2: The anchorage-independent colony formation assay in the presence of cisplatin (0.125 μM, 0.5 μM, 2 μM, 8 μM). The colony numbers (diameter >250 μm for YT-nu, >100 μm for NB39) in soft agar are shown ($n = 3$). $^{\#}$, The IC50 for NCAN was calculated tentatively as 8 μM, because the viability of 8 μM (highest concentration) was more than 50%.

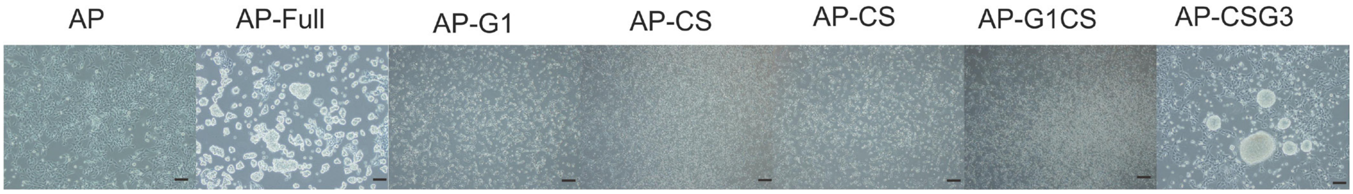

Supplementary Figure 3: Representative images of TNB1 cells treated with conditioned media containing each version of AP-fused NCAN summarized in Figure 4E. Scale bars: 100 μM.

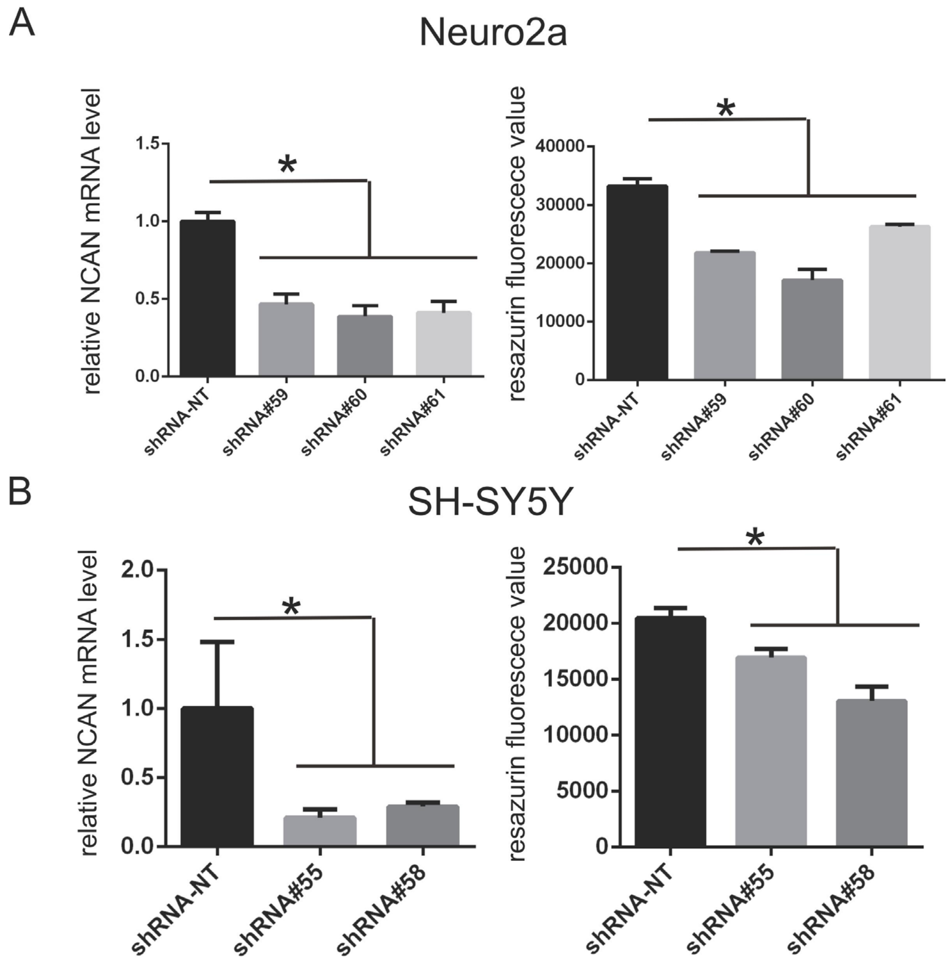

Supplementary Figure 4: The knockdown of NCAN in adherent NB cell lines inhibit their proliferation. (**A**) The knockdown of NCAN in mouse Neuro2a cells. RT-qPCR for NCAN to make sure the knockdown results by three different shRNAs (left). The resazurin fluorescence values (right). (**B**) The same patterns of experiments as (A) using human SH-SY5Y cells. $^{*}p < 0.01$.

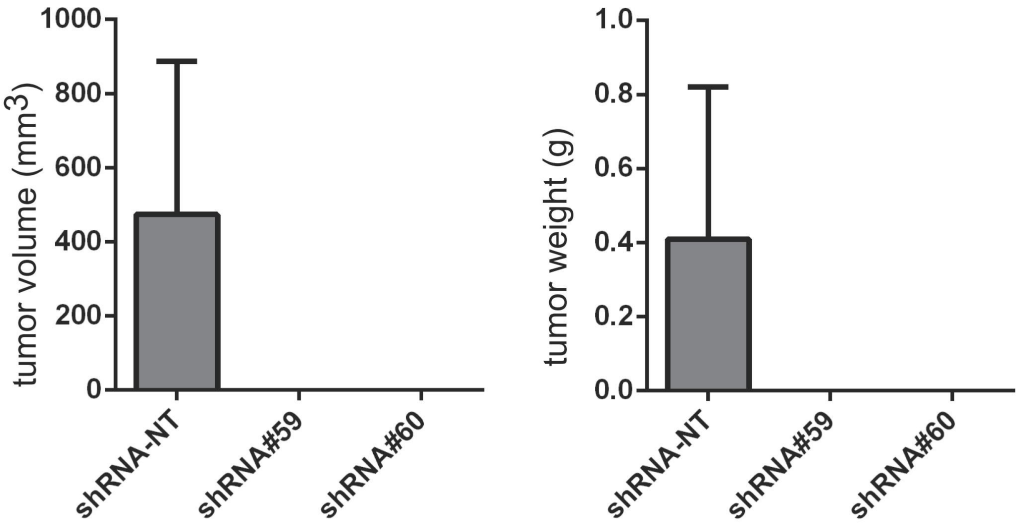

Supplementary Figure 5: Quantification of allograft tumor volumes (left) and weights (right) shown in Figure 6G.
